# Fucoidan Supplementation Restores Fecal Lysozyme Concentrations in High-Performance Athletes: A Pilot Study

**DOI:** 10.3390/md18080412

**Published:** 2020-08-04

**Authors:** Amanda J. Cox, Allan W. Cripps, Phillipa A. Taylor, J. Helen Fitton, Nicholas P. West

**Affiliations:** 1 School of Medical Science, Griffith University, Southport, QLD 4215, Australia; n.west@griffith.edu.au; 2 Menzies Health Institute Queensland, Griffith University, Southport, QLD 4215, Australia; allan.cripps@griffith.edu.au; 3 School of Medicine, Griffith University, Southport, QLD 4215, Australia; 4 Brisbane Lions Australian Football Club, Brisbane, QLD 4215, Australia; pip@piptaylor.com; 5 Marinova Pty Ltd., Cambridge, TAS 7170, Australia; helen.fitton@marinova.com.au

**Keywords:** lysozyme, gut, mucosa, athletes, seaweed, fucoidan

## Abstract

Nutritional strategies to help promote immune competence are of particular interest for a range of population groups. This study aimed to assess the potential impacts of fucoidan, a seaweed-derived bioactive polysaccharide, on gut markers of immunity and inflammation. A group of professional team-sport athletes were selected for inclusion in the study given the recognized potential for intense physical activity to induce alterations in immune function. A retrospective analysis was performed on stored fecal samples which had been collected from professional team-sport athletes (*n* = 22) and healthy adults (*n* = 11) before and after seven days of supplementation with fucoidan (*Fucus vesiculosus*/*Undaria pinnatifida* extract, 1 g/d). Fecal concentrations of calprotectin, secretory immunoglobulin A (sIgA) and lysozyme were determined using enzyme-linked immunosorbent assays. The supplement was well tolerated by participants with no adverse events reported. At baseline, fecal lysozyme concentrations were ~73% higher in the healthy adults compared to the professional athletes (*p* = 0.001). For the professional athletes, a significant (~45%) increase in fecal lysozyme was observed following the supplementation period (*p* = 0.001). These data suggest that fucoidan supplementation may have the potential to promote the secretion of antimicrobial peptides in specific population groups and contribute to the regulation of mucosal immune health.

## 1. Introduction

Strategies to help promote immune competence remain of interest for a range of specific population groups and have frequently included the use of dietary supplements. The use of bioactive ingredients from marine-based sources for formulation as food constituents and nutritional supplements is recognized as under-explored in comparison to ingredients from traditional agricultural or synthetic sources [1]. The global market for nutraceutical-type products has been estimated to exceed $380 billion per annum by 2020 [2], and there is a critical need for further exploration of marine-based bioactives to ensure that consumer demand is met by products that are both safe and have demonstrated positive health effects.

The potential for whole seaweed consumption to have positive health effects has been reviewed previously [3]. Further, the bioactive properties of constituent compounds isolated from marine algae continues to be of interest. One such compound is fucoidan. Fucoidans are a group of high molecular weight, fucose-based polysaccharides recognized as a key component of particular brown macroalgae species [4]. The bioactive properties of fucoidan preparations have been assessed in a range of in vitro and animal models [5] and include demonstrated antimicrobial [6], antiviral [7] and anticancer [8] effects. While evidence from animal models suggests fucoidans may also possess immune-modulating effects [9,10], the ability of fucoidans to act as potential modulators of mucosal health generally, and mucosal immune function appears underappreciated and requires further investigation given (i) the fucose-based structure of fucoidans, (ii) the role of fucose as a terminal sugar in human mucin glycoproteins [11], and (iii) evidence from ex vivo tissue preparations suggesting fucose may regulate gut motility [12].

We utilized a cohort of professional team-sport athletes to explore the potential impacts of fucoidan on gut markers of immunity and inflammation. The potential for intense physical activity to induce alterations in immune function, including markers of mucosal immunity, and increase risk for upper respiratory symptoms has been of interest for several decades [13,14]. Assessment of mucosal immune competence in athletes has typically utilized saliva samples and included assessment of both innate (e.g., lactoferrin and lysozyme) and adaptive (e.g., secretory immunoglobulin A; sIgA) markers and is considered relevant to protective mechanisms in the upper respiratory tract. Indeed, associations between low mucosal (tear) lysozyme concentrations [15] and salivary IgA [16,17] and risk for upper respiratory tract infection are reported in the literature. Interestingly, assessment of immune markers from other mucosal sites has been less common, which is somewhat surprising given the growing interest in gut health in athletic populations. In this regard, high-performance and elite athletes represent a unique population for assessment of potential positive effects of fucoidan supplementation on markers of mucosal immune function.

## 2. Results

The supplement was well-tolerated by participants with no adverse events reported. At baseline, fecal lysozyme concentrations were ~73% higher in the healthy adults compared to the professional athletes (*p* = 0.001; Table 1).

**Figure 1:**
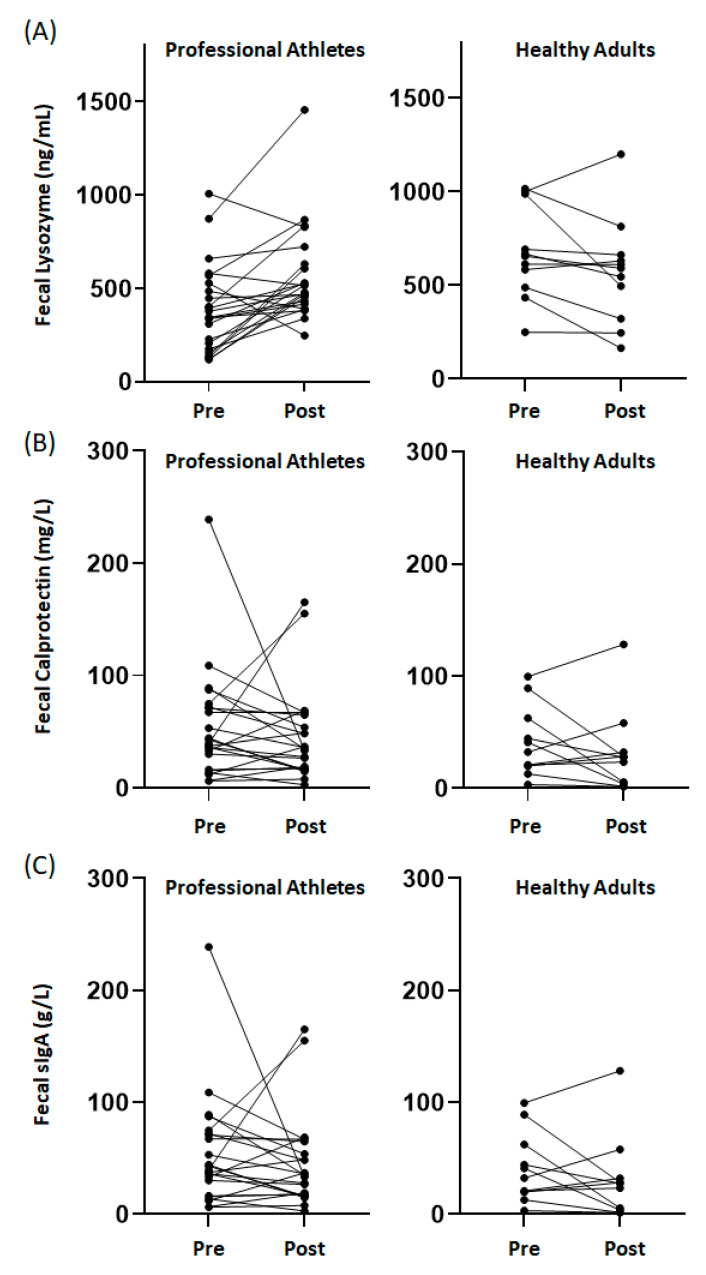
Patterns of individual response in (**A**) fecal lysozyme, (**B**) fecal calprotectin, and (**C**) fecal sIgA before (pre-) and after (post-) seven days of fucoidan in professional athletes and healthy adults.

No significant differences in baseline concentrations between groups were observed in either fecal calprotectin (*p* = 0.45) or sIgA (*p* = 0.76). Consideration of patterns of change in fecal markers (i.e., proportion of individuals showing an increase, decrease or no change in response to supplementation) in response to the seven days of supplementation suggested fecal calprotectin (*p* = 0.44) and sIgA (*p* = 0.37) responses did not differ between groups. In contrast, a different pattern of response was noted between groups for fecal lysozyme (*p* = 0.002), with a greater proportion of the professional athletes (74% of the group) showing an increase in response to supplementation when compared to the healthy adults (9% of the group). For the professional athletes, a significant (~45%) increase in fecal lysozyme was observed following the supplementation period (*p* = 0.001; Table 1). For the healthy adults, trends for reductions in both fecal calprotectin (~25%; *p* = 0.07) and lysozyme (~15%; *p* = 0.06) were observed following the supplementation period (Table 1). Individual responses are illustrated in Figure 1.

## 3. Discussion

Collectively, these data provide preliminary evidence to support the potential for fucoidan to positively impact fecal innate immune markers. Of particular note was the significant increase in fecal lysozyme concentrations in professional athletes in response to supplementation. Lysozyme is secreted from Paneth cells, in the intestinal crypts, onto the mucosal surface [18] and is excreted with the gut luminal contents allowing for determination of fecal lysozyme concentrations. Lysozyme is recognized as possessing both antimicrobial and anti-inflammatory actions [19], and evidence from animal models suggests a role for lysozyme in promoting mucosal barrier integrity [20]. Considering these functions, the increase in fecal lysozyme may reflect improvements in mucosal health. The increase in fecal lysozyme concentrations in professional athletes is also worth considering further given that lower fecal lysozyme concentrations were observed compared to the healthy adults prior to supplementation. This observation is not dissimilar to reports of impaired immune defenses (primarily at other mucosal sites) in elite athletes, including reports of decreasing salivary lysozyme in rugby players across a sporting season [21]. Considering this, the return of fecal lysozyme concentrations to levels comparable to those noted for the healthy adults suggests that further exploration of the ability of fucoidan to promote the secretion of antimicrobial peptides at mucosal surfaces in specific population groups is warranted. It is also worth mentioning that the trend for a modest (15%) reduction in lysozyme concentrations among healthy adults appears to be driven by a small number of individuals (Figure 1A). The heterogeneity in this response means it is difficult to conclude whether fucoidan supplementation would be similarly beneficial in population groups where fecal lysozyme concentrations are within adequate homeostatic norms. This should be explored in future studies.

Changes in other fecal markers were not as compelling in response to fucoidan supplementation in either group. Fecal calprotectin is used clinically as a marker of intestinal inflammation [22]. While fecal calprotectin concentrations were generally considered negative (based on a clinical threshold of 100 mg/L), suggesting a likely absence of active inflammation of the gut mucosa in the study participants, we did note a trend for reduced concentrations in the healthy adults. [22] Given the modest concentrations observed and the heterogeneity in the patterns of response between healthy adults (Figure 1B), the trend for a reduction in calprotectin in response to fucoidan supplementation needs to be confirmed in additional cohorts, including where evidence of intestinal inflammation (elevated calprotectin) exists. The potential for fucoidan supplementation to modulate intestinal inflammation has been reported previously in a murine mucositis model [23]. Similarly, reports indicating that fucoidan can enhance the expression of key intestinal tight junction proteins in an in vitro model [24], when considered in conjunction with the findings described here, support the need for further assessment of the potential for fucoidan to promote gut health, in additional to potential beneficial mucosal immune effects.

This study provides preliminary evidence suggesting that fucoidan may have the potential to promote the secretion of antimicrobial peptides at the gut mucosa and contribute to the regulation of mucosal inflammation. It is recognized that the modest sample size and lack of dietary control in the current study necessitates that these findings be confirmed in a larger controlled trial, and this further work may help to resolve the heterogeneity observed in some of the patterns of response (for example the trends for reduced fecal calprotectin and lysozyme in healthy adults following supplementation). However, the outcomes provide early evidence to suggest that fucoidan may be a supplement that could be beneficial in promoting mucosal immune competence in specific population groups.

## 4. Materials and Methods

To explore the potential impacts of fucoidan on gut markers of immunity and inflammation, we performed a retrospective analysis of stored fecal samples collected from professional team-sport athletes (*n* = 22; all male; age: 24.7 ± 4.6 years; BMI: 25.1 ± 1.2 kg/m^2^) who supplemented 1 g/d fucoidan (*Fucus vesiculosus*/*Undaria pinnatifida* extract, Marinova Pty Ltd., Cambridge, TAS, Australia) during a week-long pre-season training camp. Samples were stored at −80 degrees following collection and had been thawed once prior to the analysis described herein. Comparisons were made to accessible samples which had been obtained from otherwise healthy adults (*n* = 11; 6 females, 5 males; age: 38.8 ± 9.8 years; BMI: 22.9 ± 2.5 kg/m^2^) prior to and following one week of supplementation with fucoidan (1 g/d). The composition of the fucoidan supplement was 50.5% neutral carbohydrates, 18.6% sulfate, 7.0% counter-ions, 11.2% polyphenols and 4.6% uronic acids. This analysis was performed with the approval of the Griffith University Human Research Ethics Committee (ref #2018/923). All participants provided written informed consent prior to participation.

Assessment of fecal concentrations of calprotectin, sIgA and lysozyme were determined using commercially available enzyme-linked immunosorbent assays (ELISA; Immunodiagnostik, Bensheim, Germany) specific for assessment of fecal samples. Assays were performed according to the manufacturer’s instructions, and all samples were assessed in duplicate. Typical intra-assay variability was 4.0% for calprotectin, 4.0% for sIgA and 5.2% for lysozyme. A high-range and low-range quality control were provided for inclusion in each assay; all quality controls measured within the suggested ranges.

The impact of fucoidan on fecal markers was assessed by comparing concentrations pre- and post-supplementation. Patterns of change in each outcome measure were initially assessed in a semi-quantitative manner and classified as either an increase, decrease or no change (less than a 10% increase or decrease). Differences in patterns of response between groups were assessed using a Chi-squared test. Subsequently, the distributions of outcome measures were inspected, and data were log-transformed to approximate conditional normality. Differences between pre- and post-supplementation time-points were assessed using a paired sample *t*-test. An independent sample *t*-test was used to compare baseline (pre-supplementation) concentrations between groups. Statistical analysis was completed using SPSS Statistics version 25 (IBM Corporation, Armonk, NY, USA). Statistical significance was accepted at *p* < 0.05.

## Figures and Tables

**Table 1:** Concentrations of fecal calprotectin, secretory immunoglobulin A (sIgA) and lysozyme before (pre-) and after (post-) seven days of supplementation with fucoidan (1 g/d). Data are presented as mean ± SD.

	Pre	Post	*p*-Value
Calprotectin (mg/L)			
Professional Athletes Healthy Adults	53.5 ± 49.4 40.4 ± 31.2	45.5 ± 41.3 30.6 ± 36.6	0.36 0.07
sIgA (g/L)			
Professional Athletes Healthy Adults	2.12 ± 2.02 2.73 ± 2.85	2.64 ± 2.35 2.53 ± 1.78	0.32 0.52
Lysozyme (ng/mL)			
Professional Athletes Healthy Adults	387.5 ± 237.3 670.8 ± 246.6	562.9 ± 252.5 570.2 ± 284.6	0.001 0.06
